# Thioredoxin-1 Activation by Pterostilbene Protects Against Doxorubicin-Induced Hepatotoxicity *via* Inhibiting the NLRP3 Inflammasome

**DOI:** 10.3389/fphar.2022.841330

**Published:** 2022-04-13

**Authors:** Shiqing Tan, Jie Bai, Mingxi Xu, Longying Zhang, Ying Wang

**Affiliations:** ^1^ The Second Affiliated Hospital, Dalian Medical University, Dalian, China; ^2^ Nutrition and Food Hygiene, Dalian Medical University, Dalian, China

**Keywords:** doxorubicin, pterostilbene, Thioredoxin-1, NLRP3, hepatotoxicity

## Abstract

**Background:** Doxorubicin (DOX) has been widely used in cancer treatment. However, DOX can cause a range of significant side effects, of which hepatotoxicity is a common one, and therefore limits its clinical use. Pterostilbene (PTS) has been shown to exhibit anti-oxidant and anti-inflammatory effects in the treatment of liver diseases but whether PTS could protect against hepatotoxicity in DOX-treated mice is unknown.

**Methods:** In our study, we use C57/BL6J mice and the HepG2 cell line. We divided the mice in 4 groups: the control, the PTS treatment, the DOX treatment, and the DOX + PTS treatment group. Liver histopathology was judged by performing hematoxylin–eosin and Masson staining. Immunohistochemistry was used to perform the expression of NLRP3. The levels of serum alanine transaminase (ALT) and aspartate transaminase (AST) were evaluated. Levels of malondialdehyde (MDA), superoxide dismutase (SOD), glutathione (GSH), and DCFH-DA staining were used to evaluate the oxidative injury. Western blot and real-time PCR were applied to evaluate the expressions of proteins and mRNA. MTT was used to evaluate DOX-induced cell injury and the protective effects of PTS. Recombinant Trx-1 was used to analyze the mechanism of PTS. A TUNEL assay was used to detect apoptosis in DOX-induced HepG2 cells and the protective effects of PTS.

**Results:** PTS ameliorated DOX-induced liver pathological changes and the levels of AST and ALT. PTS also decreased the level of MDA, increased the level of SOD, GSH, and the expression of Trx-1 in DOX-treated mice. PTS decreased the levels of NLRP3 and IL-1β mRNA and the expressions of their proteins in DOX-treated mice. In addition, PTS also decreased the expression of Cleaved Caspase-3 and BAX and increased the expression of BCL-2. *In vitro*, after treatment with recombinant Trx-1, ROS and NLRP3 inflammasome were both decreased. Treatment with PTS could rescue the downregulation of Trx-1, decreased the ROS level and the NLRP3 inflammasome, and protected HepG2 cells against DOX-induced apoptosis.

**Conclusion:** The results show that PTS exhibits protective effects against DOX-induced liver injuries *via* suppression of oxidative stress, fibrosis, NLRP3 inflammasome stimulation, and cell apoptosis which might lead to a new approach of preventing DOX-induced hepatotoxicity.

## Introduction

Doxorubicin (DOX), a member of the anthracycline group of structures, is used by oncologists as a highly effective drug in the treatment of tumors (Rivankar. 2014; Yang et al., 2020). However, recent studies showed that DOX causes unanticipated side-effects such as nausea, vomiting, extravasation, severe hepatotoxicity, and cardiotoxicity which limit its use in clinical practice (Carvalho et al., 2009; Kolarovic et al., 2009). The mechanism of DOX-induced hepatotoxicity is well known and is related to the generation of reactive oxygen species (ROS) that ultimately leads to cell death (Pilco-Ferreto and Calaf, 2016; Songbo et al., 2019). Therefore, targeting oxidative stress may be a therapeutic measure to rescue and prevent DOX-induced hepatotoxicity.

Pterostilbene (3,5-dimethoxy-4′-hydroxystilbene, PTS), a natural analogue of resveratrol, is a natural component of blueberries and grapes (Estrela et al., 2013; Lange and Li, 2018). PTS has many biological activities, such as an anti-oxidative, an anti-inflammatory, an anti-cancer, an anti-diabetic one etc. (McCormack and McFadden, 2012; Gómez-Zorita et al., 2021). Previous studies have demonstrated that PTS was able to significantly attenuate astrocyte inflammation and neuronal oxidative injury after ischemia-reperfusion (Liu H. et al., 2019). Sajad A Malik et al. found that PTS was able to reverse palmitic acid induced insulin resistance in HepG2 cells by reducing oxidative stress (Malik et al., 2019). Although the anti-oxidative and anti-inflammatory effects of PTS are known, the pathways leading to these effects have not yet been worked out.

Oxidative stress results from an imbalance in the number of pro-oxidant and anti-oxidant molecules. Among anti-oxidants, thioredoxin-1 (Trx-1) and nicotinamide adenine dinucleotide phosphate (NADPH) form an important and ubiquitous redox system (Powis and Montfort, 2001; Lu and Holmgren, 2014; Perkins et al., 2014; Lu et al., 2021). Trx-1 is a sulfhydryl disulfide oxidoreductase that acts as a reducing agent for oxidized proteins (Hashemy and Holmgren, 2008). The oxidized form of Trx-1 is, in turn, reduced by NADPH (Pillay et al., 2011). Several studies have confirmed that Trx-1 exerts a protective effect in liver injuries but the mechanism is still unclear (Wang X. et al., 2019). NOD-like receptors (NLR) are multi-component assemblies that, in case they are containing the pyrin domain 3, are classified as NLRP3 proteins and as such are part of the so-called inflammasome which also comprises the adapter protein apoptosis-related speck-like protein (ASC) and pro-caspase-1 (Zhang et al., 2021). NLRP3 plays an important role in inflammatory stimulation and regulation (Chen et al., 2019). Previous studies showed that Trx-1 modulates NLRP3 inflammasome activities during atherosclerosis development (Wang et al., 2020). In this study, we will try to shed some light on the presumed PTS modulation of the Trx-1/NLRP3 signaling pathway and the PTS use as a protective agent in DOX-induced hepatotoxicity.

## Materials and Methods

### Chemicals

PTS (purity >99%) was purchased from Meilunbio (Dalian, Liaoning Province, China). DOX was purchased from Sigma-Aldrich (St. Louis, MO, United States). Alanine transaminase (ALT) and aspartate transaminase (AST) were from Nanjing Jiancheng Institute of Biotechnology (Nanjing, China). Malondialdehyde (MDA), superoxide dismutase (SOD), and glutathione (GSH) kits were purchased from Solarbio (Beijing, China). Hematoxylin–eosin (H & E) and Masson staining kits were from Beyotime Biotechnology (Shanghai, China). 3-(4,5-Dimethylthiazol-2-yl)-2,5-diphenyltetrazolium bromide (MTT) was provided by Roche Diagnostics (Basel, Switzerland). TUNEL staining kits (Green) were from Beyotime Biotechnology. The bicinchoninic acid (BCA) protein assay kit was from Thermo Scientific, lysis buffer and phenylmethanesulfonylfluoride (PMSF) were obtained from Beyotime Biotechnology. Human recombinant Trx-1 was from Med Chem Express (HY-P73431).

### Animals and Treatment

We used 8-week-old wild type (WT) C57/BL6J mice as experimental animals and divided the mice into four groups (with *n* = 8 for each group): a control group, a PTS treatment group, a DOX treatment group, and a DOX + PTS treatment group. The animals of the DOX group were injected a dose of 10 mg/kg intraperitoneally. This was conducted on day 1 and day 4 for a total of 2 times (20 mg/kg cumulative dose of DOX). The mice of the DOX + PTS group were injected intraperitoneally with PTS (10 mg/kg/day) every day for a total of 7 times, until one day before DOX treatment. As in the DOX treatment group, afterwards DOX was injected intraperitoneally with a dose of 10 mg/kg. This was conducted on day 1 and day 4 for a total of 2 times (20 mg/kg cumulative dose of DOX). All mice were euthanized 6 days after the initial injection of DOX (Liu D. et al., 2019). All the animal experiments were approved by the Institutional Animal Care and Use Committee of the University of Dalian Medical University (SCXK 2015-2003).

### Histopathology and Immunohistochemical Staining

All the mice were sacrificed under anesthesia after our study period. The liver tissue was fixed with 4% paraformaldehyde (PFA) for more than 24 h, followed by paraffin embedding. All sections (4 μm) were subjected to a H & E and Masson staining. The liver tissues were subjected to immunohistochemical staining. For this, the sections were incubated with the primary antibody anti-NLRP3 (Wanleibio, WL02635, 1:200) at 4°C overnight and afterwards with the corresponding secondary antibody. The blots were developed using DAB. Digital images were taken at 200 × magnification and were analyzed with ImageJ software.

### Measurements of MDA, SOD and GSH Levels

The levels of MDA, SOD, and GSH in DOX-treated livers were evaluated by MDA, SOD, and GSH kits (Solarbio), respectively, according to the manufacturer’s instructions.

### Cell Culture and Experiments

HepG2 cell were purchased from the American Type Culture Collection. Cells were cultured in DMEM supplemented with 10% fetal bovine serum and antibiotics (100 U/ml penicillin and 100 μg/ml streptomycin, Sigma) and were grown in a humidified atmosphere containing 5% CO_2_ at 37°C. Hep G2 cells were treated with recombinant Trx-1 at a dose of 1 μg/ml to elucidate the presumed PTS effects (El Hadri, K., et al., 2012).

### DOX-Induced Cell Injury

HepG2 cells were seeded in 96-well plates for 24 h. After 24 h, the medium was removed, 100 μL of sample solution with different concentrations of DOX (0, 1, 2, 5, 8, and 10 mM) was added for different treatment times and a period of 24 h (Song, et al., 2019b). MTT solution (5 mg/ml) was added to each well to a final concentration of 0.5 mg/ml for 4 h. After exposure, DMSO (100 μL/well) was added to dissolve the formed formazan crystals. The absorbance at 490 nm was measured with a microplate reader (Thermo, United States). Based upon these data, a suitable DOX concentration for the induction of cell injury was identified.

### Cell Viability Assay

HepG2 cells were seeded in 96-well plates for 24 h and then pretreated with different concentrations of PTS (0, 5, 10, and 20 μM) for 16 h before the treatment with DOX (5 μM) for 24 h. DOX group cells were cultured without PTS pretreatment. An MTT assay was used to assess cell viability (Song S. et al., 2019).

### Measurement of ROS Level in HepG2 Cells

HepG2 cells were plated in 6-well culture plates for 24 h and afterwards treated with PTS at a concentration of 10 μM for 4 h before treatment with DOX (5 μM) for 24 h (Song S. et al., 2019). DOX group cells were cultured without PTS pretreatment and the control group was cultured in serum-free DMEM under normal conditions during the entire experiment. Cells were loaded with 10 μM DCFH-DA. After that, the cells were washed 3 times with serum-free DMEM and the images were captured by fluorescence microscopy (Olympus, Japan) with a 200 × overall magnification.

### TUNEL Assay for HepG2 Apoptosis

HepG2 cell apoptosis detection was performed by using TUNEL staining (Green, Beyotime Biotechnology) and the assay was performed according to the manufacturer’s instructions. For this, HepG2 cells were plated in 6-well culture plates for 24 h and afterwards treated with PTS at a concentration of 10 μM for 4 h before the treatment with DOX (5 μM) for 24 h. After that, the cells were washed 3 times with PBS and fixed with 4% PFA for 30 min after which the cells were washed 3 times with PBS. Afterwards, the cells were treated with 0.3% Triton X-100 containing PBS solution for 5 min, washed with PBS 3 times and finally the cells were incubated with the TUNEL reaction mixture for 60 min at 37°C in the dark. The cells were evaluated under a fluorescence microscope.

### Biochemical Analysis

All mice were sacrificed under anesthesia after our study period. The serum of the animals was collected, and AST and ALT were measured by employing commercially available biochemical kits that were used according to the manufacturer’s instructions.

### Western Blot Assay

Total proteins were extracted from snap-frozen liver tissues or cells. We use a protein extraction kit (Keygenbio, KGP250) and centrifuge tube (Guangzhou Jet Bio-Filtration Co., Ltd.) for proteins extracting. The protein lysates (30 μg) were separated by electrophoresis in an 8–15% SDS–PAGE gel and transferred to polyvinylidene difluoride (PVDF) membranes. The blots were incubated with appropriate antibodies at 4°C overnight and then incubated with a goat anti-rabbit or mouse conjugated secondary antibody (Sino Biological Inc., 1:3000). All blots were developed using an ECL Plus chemiluminescence system. The following antibodies were used: anti-Trx-1 (CST, #2429, 1:800), anti-NLRP3 (CST, #15101, 1:800), Caspase-1 p20 (Affinity, AF5418, 1:500), IL-1β (Wanleibio, WL0227, 1:500), IL-18 (Wanleibio, WL01127, 1:1000), ASC (Wanleibio, WL02462, 1:500), BAX (CST, #14796S, 1:500), BCL-2 (CST, #3498S, 1:500) and Cleaved Caspase-3 (CST, #9664, 1:500). ImageJ software was used for densitometry analysis and GAPDH was used as an internal control.

### Real-Time PCR Assay

According to the manufacturer’s instructions, we used TRIzol reagent (Invitrogen, New York) to purify the total RNA from the fresh livers and cells. The first-strand cDNA (1–2 μg) was synthesized using a Superscript II kit (TAKARA, Japan). All the primers were synthesized by Sangon Biotech Company (Shanghai, China). The primer sequences were as follows: NLRP3: forward 5′-AGC CAA GAA TCC ACA GTG TAA CC-3′ and reverse 5′-AGT GTT GCC TCG CAG GTA AG-3′; IL-1β: forward 5′-TGC CAC CTT TTG ACA GTG ATG-3′ and reverse 5′-TTC TTG TGA CCC TGA GCG AC-3′; IL-18: forward 5′- GCA AAG CTT ATG ACC ATG AGA CAC AAC TG-3′ and reverse 5′-GCG AAT TCG TCG ACT TTA ACC CTG CTG TGG ACT-3′; NOX-1: forward 5′-GCT ACG CCT TCA ACA CCA AG-3′ and reverse 5′-AGT TCG TCC CCT TCT CCT GT-3′; NOX-4: forward 5′-GCA CGC TGT TGA TTT TTA TGG-3′ and reverse 5′-GCG AGG CAG GAG AGT CAG TA-3′; GAPDH: forward 5′-CAT CAA GAA GGT GGT GAA-3′ and reverse 5′-TGT TGA AGT CAG AGG AGA-3′. We used GAPDH as the internal control and normalized the resulting transcript levels to those of GAPDH gene. The results were analyzed using the ΔΔCt technique.

### Statistics

All data are expressed as the mean ± SD. The statistical analyses were performed with GraphPad Prism 9 software. One-way ANOVA followed by Tukey’s comparison test was used to analyze significant differences among multiple groups. Values of *p* < 0.05 were considered as being statistically significantly different.

## Results

### Treatment with PTS Suppresses DOX-Induced Hepatotoxicity, Fibrosis and Oxidative Stress Injury in Mice

**FIGURE 1:**
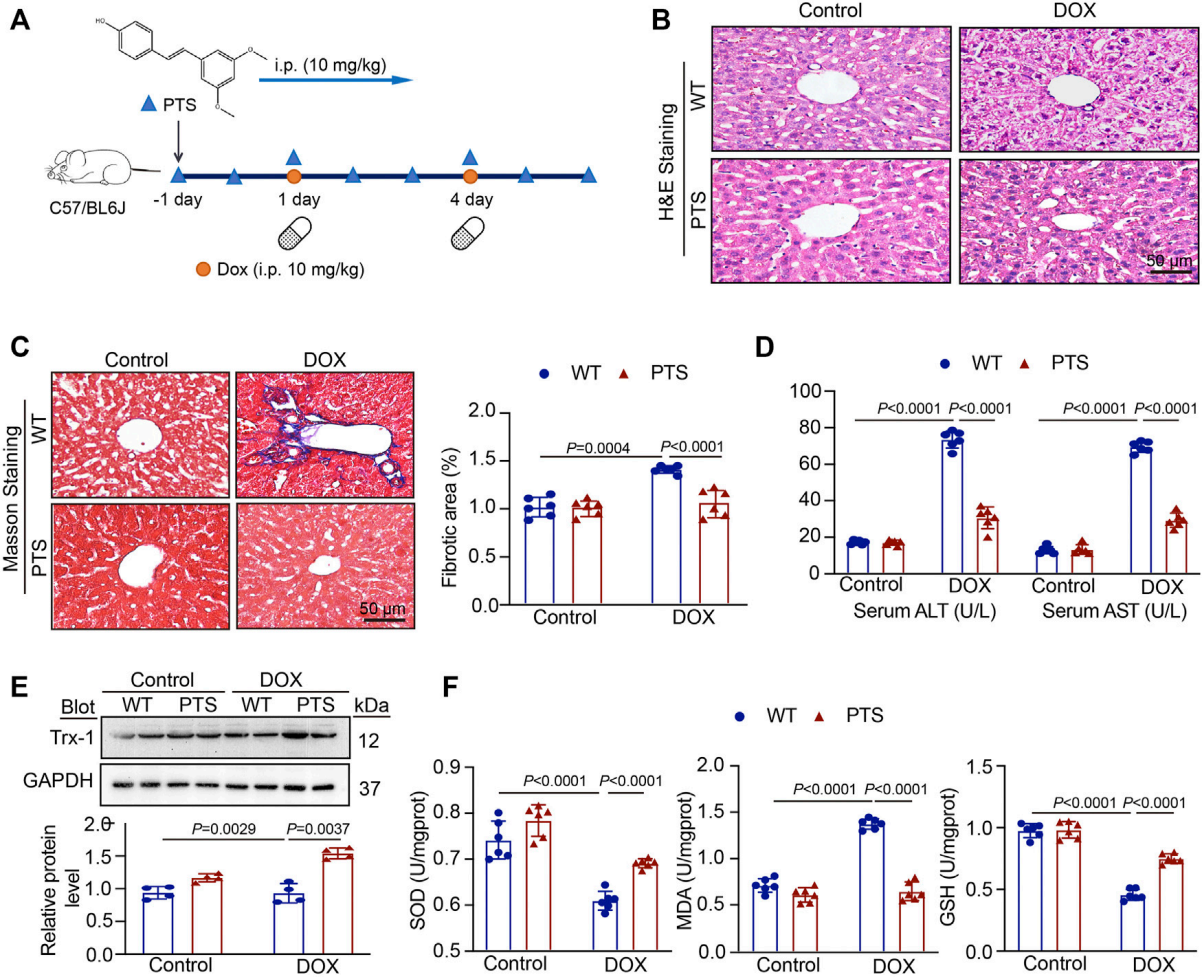
PTS treatment prevents DOX-induced hepatotoxicity. **(A)** Diagrammatic representation of different mice treatments: PTS (10 mg/kg) was injected every day for a total of 7 times (one day before DOX treatment); DOX administration was conducted on day 1 and day 4 for a total of 2 times (20 mg/kg cumulative dose of DOX); **(B)** H & E staining of each group were analyzed (scale bar = 50μm, *n* = 6 per group); **(C)** Masson staining of each group was analyzed (left, scale bar = 50 μm), the quantification of the fibrotic area (right, *n* = 6); **(D)** The levels of serum ALT (left) and AST (right) in each group (*n* = 6 per group); **(E)** Western blot analysis of Trx-1 protein in each group (up), the quantification of Trx-1 expression (down, *n* = 4 per group); **(F)** The levels of SOD (left), MDA (middle), and GSH in each group (*n* = 6 per group).

To explore the effects of PTS on DOX-induced hepatotoxicity, we pre-treated the mice with PTS (10 mg/kg) before DOX administration (Figure 1A). H & E staining (Figure 1B) revealed that the liver of control group mice displayed a normal architecture whereas apparent injuries were found in the DOX-treated group that could be restored by PTS. In addition, Masson staining revealed that administration of DOX in mice markedly increased the collagen deposition compared to the control group and that PTS remarkably reduced the DOX-induced fibrosis (Figure 1C). As shown in Figure 1D, after treatment with DOX, compared with the control group, the levels of ALT and AST were increased, respectively. The pre-treatment of PTS significantly reduced the ALT and AST levels in mice compared to the DOX-treated group. We next detected the expression of Trx-1 protein. Compared to the control group, the expression of Trx-1 was obviously decreased after DOX treatment but could be rescued by PTS (Figure 1E). We next evaluated the SOD, GSH, and MDA levels in DOX-treated mice. As shown in Figure 1F, the SOD and GSH levels were both markedly decreased after treatment with DOX compared to the control group and the MDA level in the DOX-treated group was higher than that of the control group. After PTS treatment, the level of MDA was decreased and the levels of SOD and GSH were both increased compared to the DOX-treated group. Therefore, PTS was able to prevent the increase in ROS and the decrease of Trx-1 in DOX-treated mice.

### PTS Application Alleviates the Inflammation Reaction and Cell Apoptosis in DOX-Treated Mice

**FIGURE 2:**
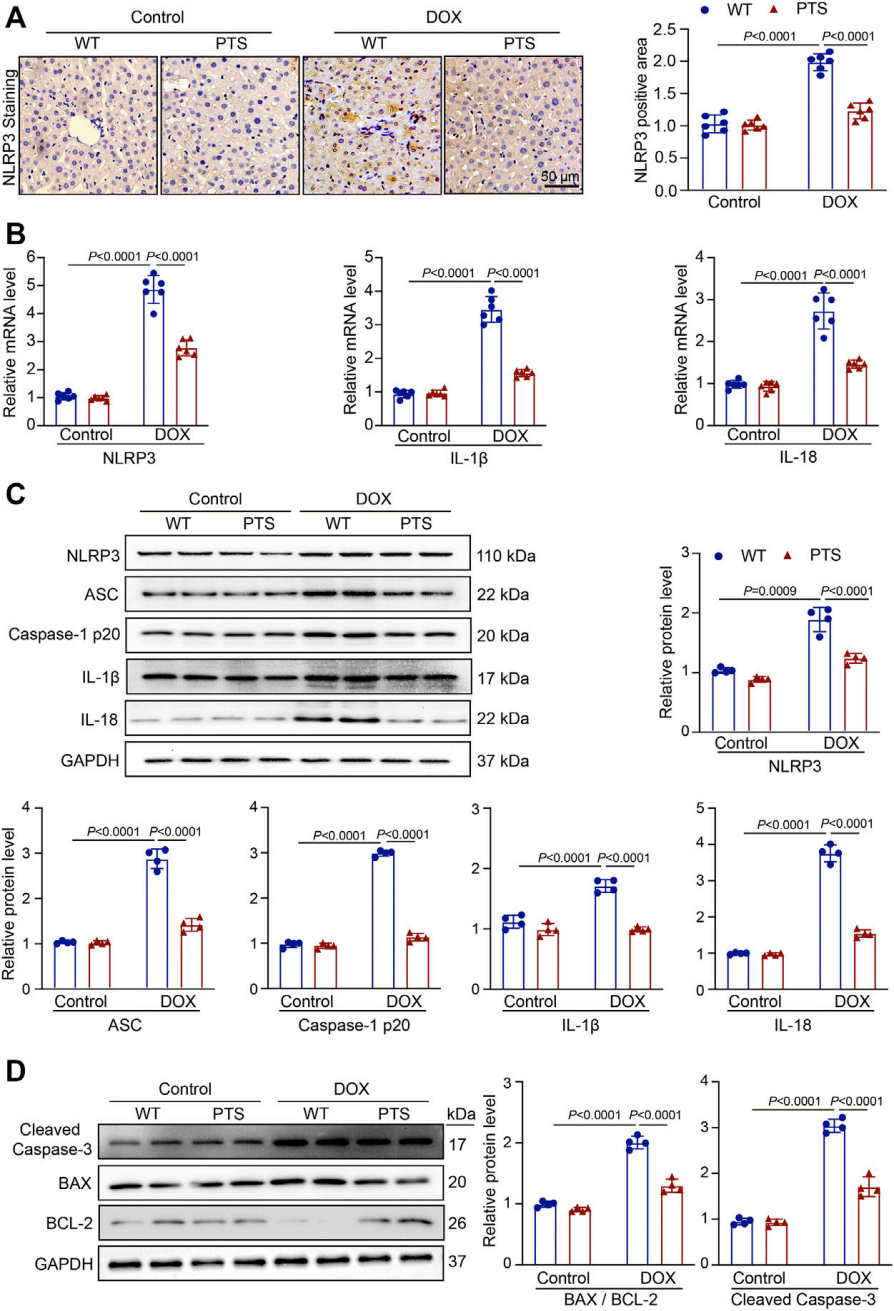
PTS reduces reduction of NLRP3 inflammasome and apoptosis in DOX-treated mice. **(A)** WT C57/BL6J mice were pretreated with PTS (10 mg/kg) and afterwards with DOX (20 mg/kg cumulative dose) for 6 days. Immunochemistry staining of the liver sections with anti-NLRP3 (left, scale bar = 50 μm), the quantification of NLRP3 positive area in each group (right, *n* = 6); **(B)** qPCR analyses of NLRP3, IL-1β and IL-18 mRNA levels in each group (*n* = 6); **(C)** Western blot analyses of NLRP3, ASC, Caspase-1 p20, IL-1β, and IL-18 proteins and the quantification of the blots in each group (*n* = 4 per group); **(D)** Western blot analyses of Cleaved Caspase-3, BAX, and BCL-2 proteins (left) and quantification of the blots in each group (right, *n* = 4 per group).

Oxidative stress frequently results in inflammatory reactions (Ventura et al., 2017). To further elucidate the effects of PTS, we performed immunohistochemical staining to detect the expression of NLRP3 in DOX-treated mice. As shown in Figure 2A, the expression of NLRP3 was upregulated in the DOX-treated group and PTS was able to alleviate the increase in NLRP3 expression. We then evaluated the levels of NLRP3, IL-1β, and IL-18 mRNA. As shown in Figure 2B, PTS significantly reduced the levels of NLRP3, IL-1β and IL-18 mRNA compared to those in the DOX-treated group. In addition, we evaluated the expressions of NLRP3 and its downstream proteins. Compared to the DOX-treated group, the expression of NLRP3, ASC, Caspase-1 p20, IL-1β, and IL-18 was significantly decreased after pretreatment with PTS (Figure 2C). In addition, we detected the expressions of Cleaved Caspase-3, BAX, and BCL-2 proteins. As shown in Figure 2D, the expression of Cleaved Caspase-3 and BAX were increased, and the expression of BCL-2 was significantly decreased after treatment with DOX. Compared to the DOX-treated mice, the pretreatment of PTS reduced the expression of BAX and upregulated the expression of BCL-2.

### PTS Pretreatment Rescues DOX-Induced Cell Viability Inhibition

**FIGURE 3:**
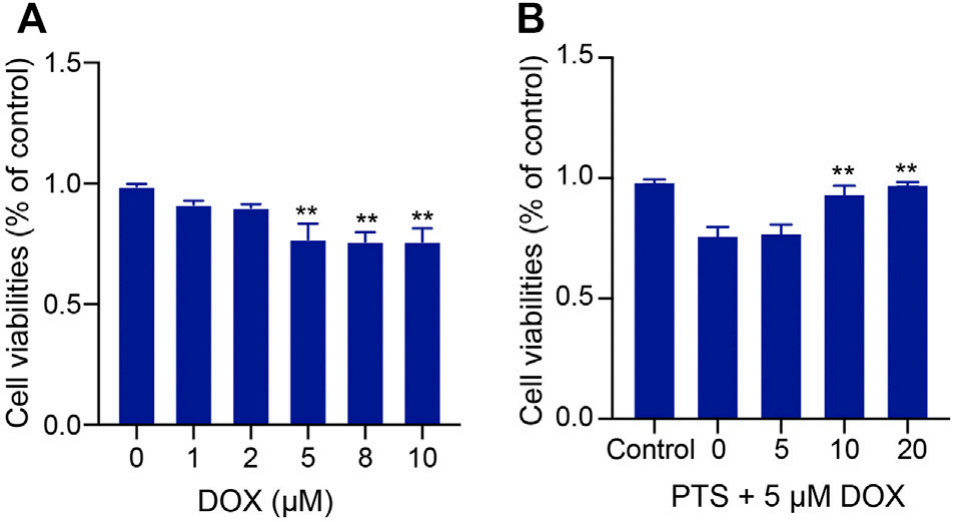
Effect of PTS treatment on cell viability in DOX-treated HepG2 cells. **(A)**. Effects of different DOX dose (0, 1, 2, 5, 8, 10 μM) - induced hepatotoxicity on HepG2 cells (*n* = 3); **(B)** PTS effects (0, 5, 10, and 20 μM) on DOX-induced HepG2 cell viability (*n* = 3). **p < 0.01 versus control group.

HepG2 cells were treated with different DOX doses (0, 1, 2, 5, 8, 10 μM) for 24 h. As shown in Figure 3A, the viability of HepG2 cells treated with 5 μM DOX for 24 h was decreased to nearly 75%, which is why we treated the cells in the following experiments with 5 μM DOX. We used PTS at concentrations of 0, 5, 10, and 20 μM to check whether PTS could protect cells against DOX-induced injury in a dose dependent manner. HepG2 cells were pretreated with different PTS concentrations for 4 h and afterwards treated with DOX (5 μM) for 24 h. Compared to the DOX-treated group, PTS at a 10 and 20 μM concentration significantly increased the viability of HepG2 cells (Figure 3B) so that we treated the cells in the following experiments with a PTS concentration of 10 μM.

### DOX Induces Inflammation in HepG2 Cells by Reducing the Trx-1 Expression

**FIGURE 4:**
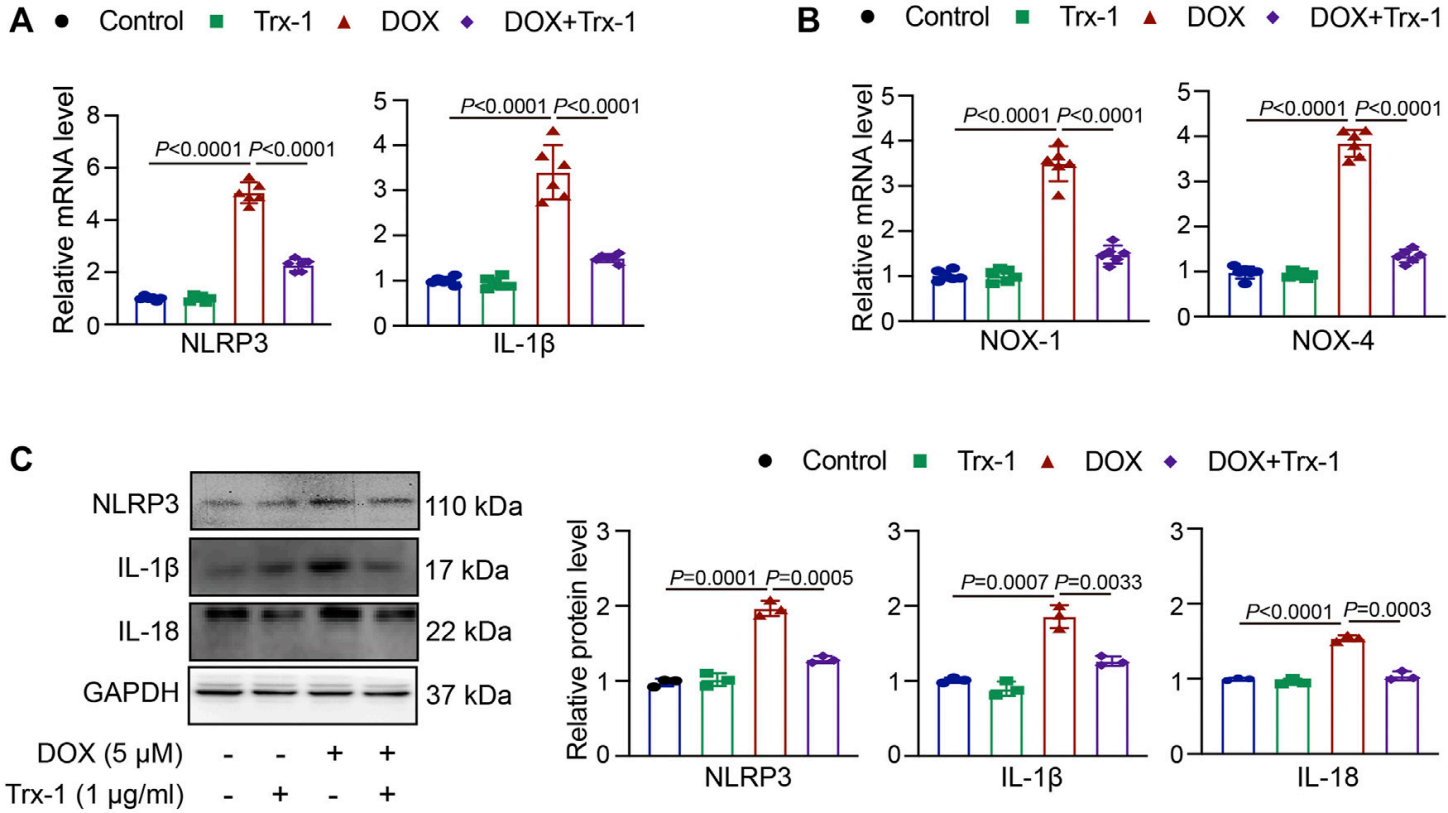
DOX-induced damage in HepG2 cells through Trx-1/NLRP3 signaling. **(A)** HepG2 cells were pretreated with recombinant Trx-1 (1 μg/ml) for 4 h afterwards with DOX (5 μM) for 24 h qPCR analyses of NLRP3 and IL-1β mRNA levels after application of recombinant Trx-1 in DOX-treated cells (*n* = 6); **(B)** qPCR analyses of NOX-1 and NOX-4 mRNA levels after application of recombinant Trx-1 in DOX-treated cells (*n* = 6); **(C)** Western blot analyses of NLRP3, IL-1β, and IL-18 protein expressions after application of recombinant Trx-1 in DOX-treated cells (left, *n* = 3), the quantification of NLRP3, IL-1β, and IL-18 protein expressions (right, *n* = 3).

To clarify whether the DOX-induced upregulation of NLRP3 was mediated by the reduction in Trx-1 levels, we pretreated the HepG2 cells with recombinant Trx-1 (1 μg/ml) for 4 h and afterwards with DOX (5 μM) for 24 h. Upon administration of recombinant Trx-1, the levels of both NOX-1 and NOX-4 were decreased compared to those of the DOX-treated group (Figure 4A). As shown in Figure 4B, this was also true for the NLRP3 and IL-1β mRNA levels. Similarly, the expressions of NLRP3, IL-1β, and IL-18 protein were decreased (Figure 4C). The results confirmed that the upregulation of Trx-1 was able to decrease the NLRP3 signal and inflammasome stimulation.

PTS inhibits DOX-induced oxidative stress in cells through increasing Trx-1 expression.

**FIGURE 5:**
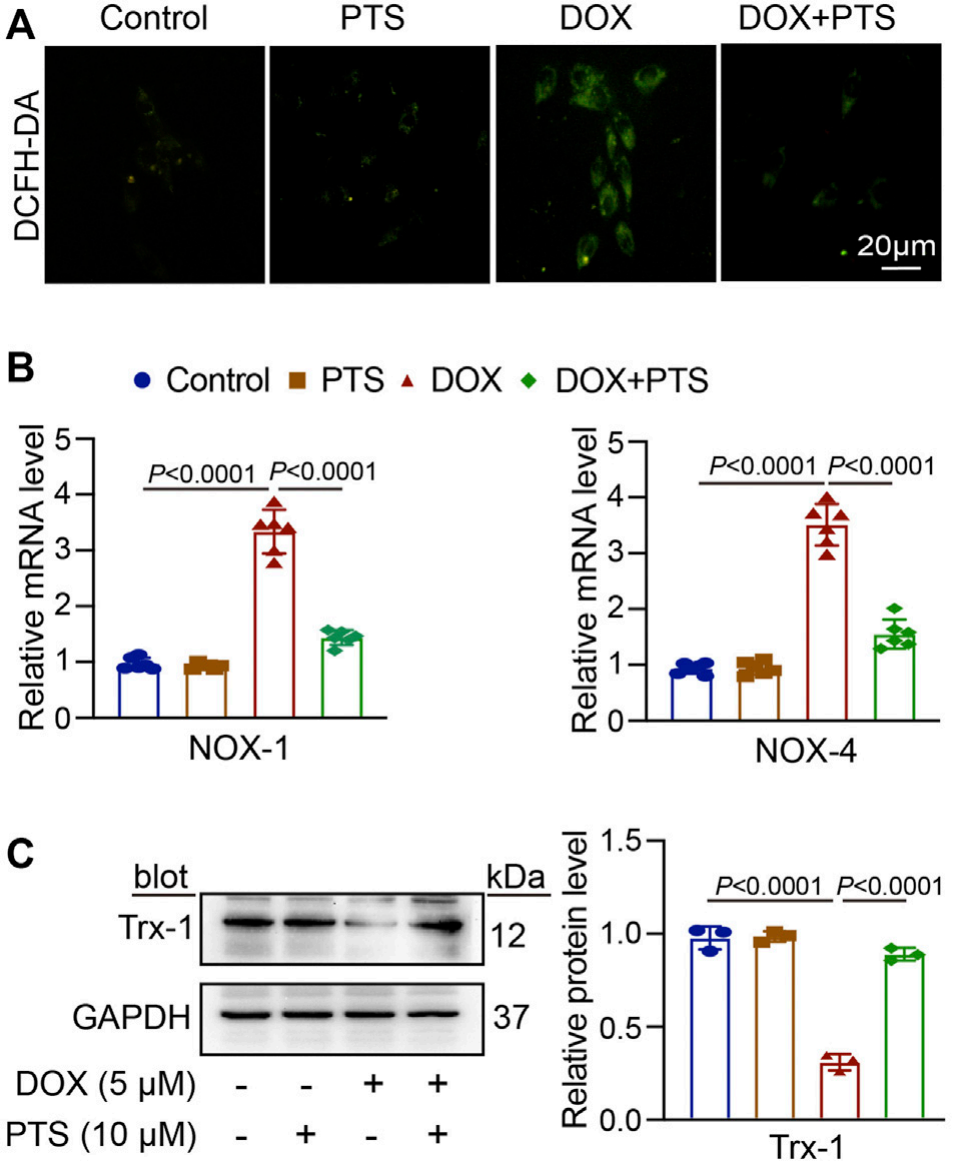
PTS increases the expression of Trx-1 and prevents oxidative damage in DOX-treated HepG2 cells. **(A)** HepG2 cells were pretreated with PTS (10 μM) for 4 h and afterwards with DOX (5 μM) for 24 h. DCFH-DA staining of each group was analyzed to detect possible effects of PTS on the ROS level in DOX-treated HepG2 cells (*n* = 3); **(B)** PTS effects on NOX-1 and NOX-4 mRNA levels in DOX-treated HepG2 cells (*n* = 6); **(C)** PTS effects on Trx-1 protein expression in DOX-treated HepG2 cells (left, *n* = 3), quantification of Trx-1 protein expression (right, *n* = 3).

We used a DCFH-DA staining to detect the effect of PTS on DOX-treated cells. HepG2 cells were pretreated with PTS (10 μM) for 4 h and afterwards treated with DOX (5 μM) for 24 h. As shown in Figure 5A, treatment with DOX increased the ROS level in HepG2 cells compared to the control group. After pretreatment with PTS, the cellular ROS levels were significantly decreased compared to those in the DOX-treated group. Moreover, after treatment with DOX, the NOX-1 and NOX-4 mRNA levels were both increased compared to the control group. After pretreatment with PTS, NOX-1 and NOX-4 mRNA levels were decreased compared to those in the DOX-treated group (Figure 5B). We next analyzed the expression of Trx-1. As shown in Figure 5C, DOX induced the downregulation of Trx-1 and PTS pretreatment was able to rescue the expression of Trx-1.

### PTS Relieves the Inflammatory Reaction in DOX-Treated Cells

**FIGURE 6:**
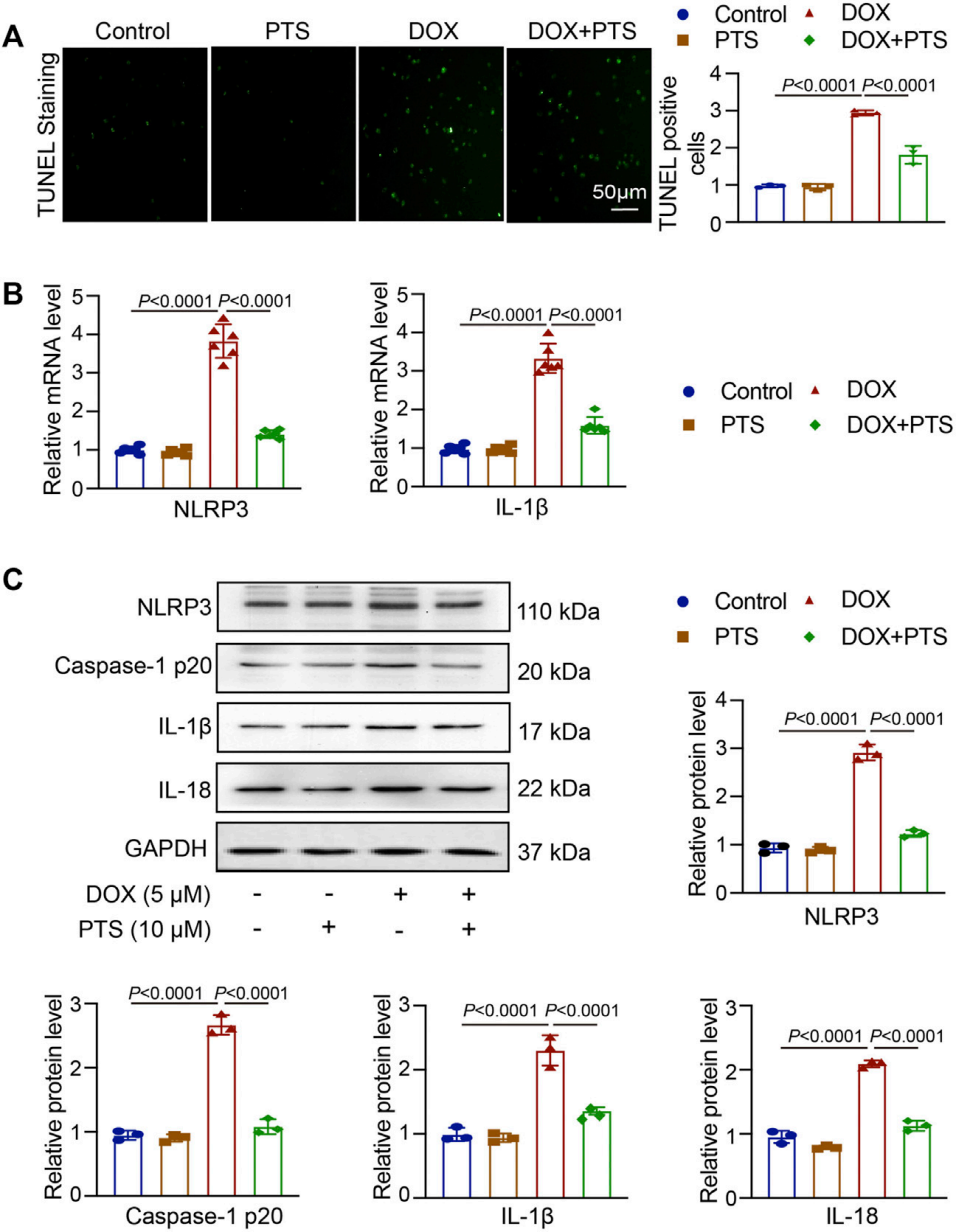
PTS effects on NLRP3 inflammasome expression in DOX-treated HepG2 cells. **(A)** HepG2 cells were pretreated with PTS (10 μM) for 4 h and afterwards with DOX (5 μM) for 24 h. The cells were analyzed by TUNEL staining to detect apoptosis in DOX-treated HepG2 cells and the protective effects of PTS against DOX-induced apoptosis (left), relative quantification of TUNEL positive cells (right, *n* = 3); **(B)** PTS effects on NLRP3 and IL-1β mRNA levels in DOX-treated HepG2 cells (*n* = 6); **(C)** PTS effects on NLRP3, IL-1β, and IL-18 protein expression in DOX-treated HepG2 cells (*n* = 3); quantification of NLRP3, IL-1β, and IL-18 protein expressions (*n* = 3).

A TUNEL assay was used to detect apoptosis in DOX-treated HepG2 cells and the protective effects of PTS. For this, HepG2 cells were pretreated with PTS (10 μM) for 4 h whereafter they were treated with DOX (5 μM) for 24 h. As shown in Figure 6A, DOX induced HepG2 cell apoptosis which could be reverted by pretreatment with PTS. Compared to the control group, the NLRP3 and IL-1β mRNA levels were markedly increased after DOX treatment and this effect was inhibited by PTS. Similarly, the expression of NLRP3, Caspase-1 p20, IL-1β, and IL-18 were significantly decreased after PTS treatment (Figure 6B).

## DISCUSSION

DOX is a potent anti-cancer agent and has been widely used in chemotherapeutic treatment regimens against breast, gastric, thyroid, lung, and ovarian cancers (Pugazhendhi et al., 2018; Tacar et al., 2013). However, DOX may cause a range of significant side effects in normal tissues one of which is hepatotoxicity (Prathumsap et al., 2020; Wang et al., 2010; Prasanna et al., 2020; Ingawale et al., 2014; Pingili et al., 2019). Several studies have shown that the protective effect of anti-oxidant agents against DOX-induced hepatotoxicity is mediated *via* regulatory mechanisms related to oxidative stress and inflammation (Jeon et al., 2014; Wang R. et al., 2019). PTS is a natural stilbene derived from resveratrol that displays a higher oral bioavailability and bioactivity but is far less abundant in natural sources (Liu H. et al., 2019). The molecule exerts diverse pharmacological activities, comprising anti-oxidation and anti-inflammation effects (Song L. et al., 2019). Previous studies showed that PTS was able to prevent hepatocyte epithelial-mesenchymal transition in fructose-induced liver fibrosis through modulating the Sirt1/p53 and TGF-β/Smads signaling pathway (Song et al., 2019). We found that a mice pretreatment with PTS was able to decrease a DOX-induced fibrosis (Figure 1C). PTS also can reverse palmitic acid mediated insulin resistance in HepG2 cells by reducing oxidative stress (Malik et al., 2019). In addition, Dong et al. have found that PTS was able to ameliorate DOX mediated cardiotoxicity by reducing oxidative stress (Liu et al., 2020).

In our study, we applied PTS to explore the protective effects in DOX-induced hepatotoxicity. Due to previous reports, we chose a single DOX dose to induce hepatotoxicity. The DOX dose (20 mg/kg) is based on the clinical data for treating cancer patients (Chen et al., 2016). Moreover, we chose a single PTS dose (10 mg/kg) to detect the protective effects on DOX-induced hepatotoxicity (Yang et al., 2016). However, due to the fact that a single dose treatment has some limitations, future experiments will evaluate the protective effect of a repeated dosage. In this study, we could show that the serum ALT and AST levels both were decreased after PTS treatment in the DOX-treated group. PTS treatment also alleviated DOX-induced histopathological changes in mice. The results imply that PTS has protective effects by inhibiting DOX-induced hepatotoxicity, however, the mechanisms are complex.

Several groups reported that DOX-induced hepatotoxicity was resulting from ROS over-production, the imbalance between pro-oxidant and anti-oxidant molecule concentrations and inflammation over-activation (Lu and Holmgren, 2014). Trx-1 is an evolutionarily conserved protein disulfide reductase. Using two cysteines at catalytic centers 32 and 35, Trx-1 cuts the disulfide bonds of oxidized proteins and forms disulfide bonds in Trx-1 (Oka et al., 2020). Trx-1 has been considered as an important protective system against oxidative stress (El Hadri et al., 2012) and is also involved in controlling inflammatory responses (Ito et al., 2011). The NLRP3 inflammasome is a multi-component assembly composed of NLRP3, ASC, and Caspase-1 precursor (Zhang et al., 2021) and has been reported to be involved in the pathogenesis of liver injury (Iskusnykh et al., 2021). Trx-1 could inhibit the NLRP3 inflammasome leading to an attenuation of atherosclerosis and was able to exert protective effects (Wang et al., 2020). In our study, we found that after DOX treatment, the SOD and GSH levels were both markedly decreased, whereas the MDA level was increased, both of which were ameliorated by PTS in mice livers. We next measured the expression of Trx-1, the results showed that DOX treatment downregulated Trx-1 expression and that PTS could recuperate Trx-1 expression (Figure 1). Therefore, in our study, we next tested the expression of NLRP3 and its downstream proteins ASC, Caspase-1, IL-1β, and IL-18. The results showed that DOX upregulated the expression of NLRP3 inflammasome and that PTS decreased its stimulation (Figure 2). Consequently, inhibiting oxidative stress and inflammation reactions by influencing the Trx-1/NLRP3 signaling pathway might be the way how PTS is able to reduce the DOX-induced hepatotoxicity. Recent studies have found that Trx-1 was able to inhibit apoptosis through redox regulation and inflammation (Bai et al., 2021). We, therefore, evaluated the expression of Cleaved Caspase-3, BAX, and BCL-2 proteins and found PTS was able to protect mice against DOX-induced apoptosis. The results confirmed that PTS could increase the expression of Trx-1 leading to a decreased ROS level and a stimulation of the inflammasome which in turn inhibited apoptosis in DOX-treated mice (Figures 1, 2).

**FIGURE 7:**
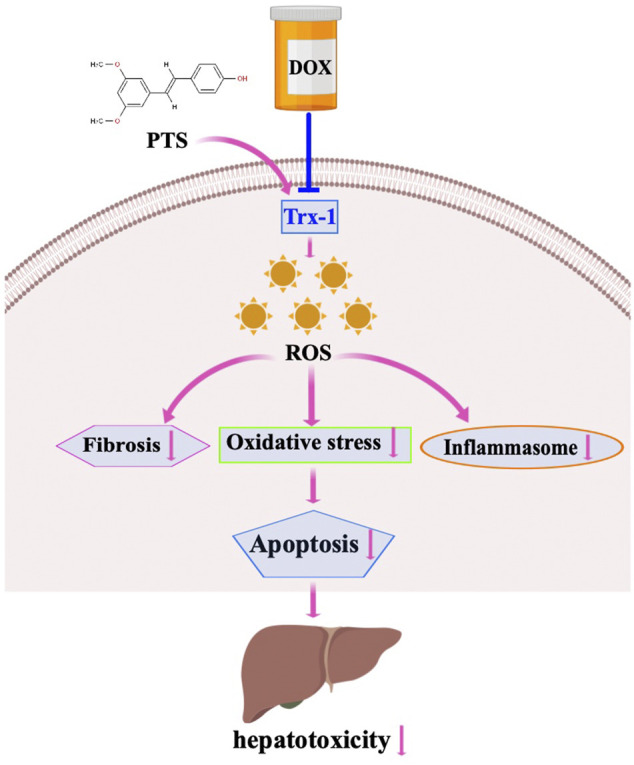
Working model for PTS in the regulation of DOX-induced hepatotoxicity. DOX treatment induced fibrosis, oxidative stress and inflammasome stimulation which resulted in hepatotoxicity through downregulation of Trx-1. PTS is able to reduce fibrosis, oxidative stress, and inflammasome stimulation through increasing Trx-1 levels. PTS may be used as an agent to protect against DOX-induced hepatotoxicity.

To further confirm the mechanisms and the protective effects of PTS in DOX-induced liver damage, we treated HepG2 cells with recombinant Trx-1. The results suggest that the Trx-1 overexpression can significantly decrease the oxidative injury and to modulate the expression of the NLRP3 inflammasome (Figure 3). PTS pretreatment has similar effects to recombinant Trx-1 - they both raised the expression of Trx-1 in DOX-treated cells. The upregulation of Trx-1 led to a reduction of ROS production, inflammasome, and apoptosis (Figures 5, 6). Taken Together, the protective effects of PTS in DOX-induced hepatotoxicity might be attributable to its anti-oxidative, anti-inflammatory, anti-fibrotic, and anti-apoptotic effects mediated by an increase in the expression of Trx-1 and activation of the Trx-1/NLRP3 signaling pathway (Figure 7). However, the detailed mechanism of PTS action in DOX-induced hepatotoxicity and its clinical application requires further studies. In conclusion, these results might point into a new direction in the therapy of this disease.

## Data Availability

The original contributions presented in the study are included in the article/Supplementary Material, further inquiries can be directed to the corresponding authors.
