# Supplementary material for: Thioredoxin-1 Activation by Pterostilbene Protects Against Doxorubicin-Induced Hepatotoxicity via Inhibiting the NLRP3 Inflammasome
Source: Front Pharmacol. 2022 Apr 13;13:841330. doi: 10.3389/fphar.2022.841330 (PMC9043100; doi:10.3389/fphar.2022.841330)

Figure 1 B H&E staining


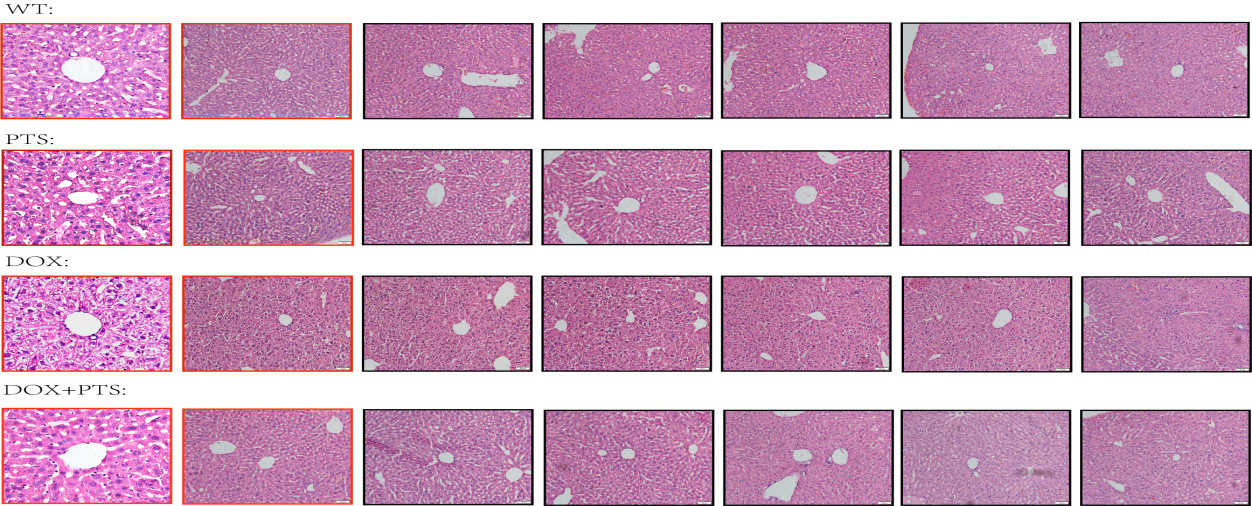


Figure 1 C Masson staining


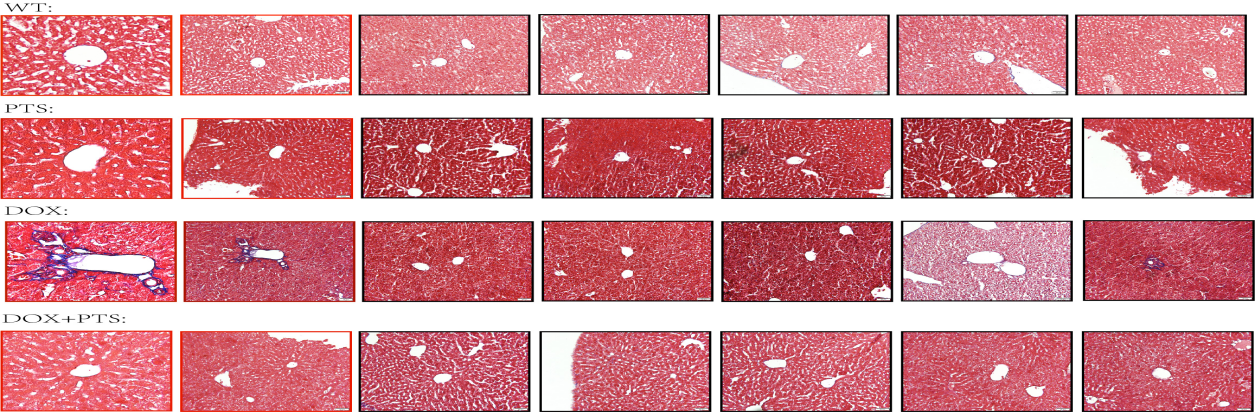


| WT | PTS | DOX | DOX+PTS |
| --- | --- | --- | --- |
| \| 1.092 \| \| --- \| \| 0.987 \| \| 0.866 \| \| 1.135 \| \| 1.018 \| \| 0.912 \| | \| 0.934 \| \| --- \| \| 0.876 \| \| 1.045 \| \| 1.034 \| \| 1.102 \| \| 1.011 \| | \| 1.433 \| \| --- \| \| 1.327 \| \| 1.379 \| \| 1.402 \| \| 1.433 \| \| 1.397 \| | \| 1.145 \| \| --- \| \| 1.166 \| \| 1.211 \| \| 0.988 \| \| 0.876 \| \| 0.917 \| |

Figure 1 D ALT AST

| WT | PTS | DOX | DOX+PTS |
| --- | --- | --- | --- |
| \| 15.44 \| \| --- \| \| 18.02 \| \| 16.73 \| \| 15.24 \| \| 17.65 \| \| 16.47 \| | \| 15.42 \| \| --- \| \| 14.18 \| \| 16.87 \| \| 16.79 \| \| 17.18 \| \| 17.97 \| | \| 76.32 \| \| --- \| \| 65.14 \| \| 77.85 \| \| 72.13 \| \| 70.43 \| \| 75.49 \| | \| 27.83 \| \| --- \| \| 19.47 \| \| 32.44 \| \| 30.18 \| \| 33.49 \| \| 36.57 \| |

| WT | PTS | DOX | DOX+PTS |
| --- | --- | --- | --- |
| \| 15.77 \| \| --- \| \| 10.34 \| \| 12.18 \| \| 13.07 \| \| 12.11 \| \| 10.45 \| | \| 10.87 \| \| --- \| \| 11.67 \| \| 10.45 \| \| 17.67 \| \| 13.11 \| \| 12.04 \| | \| 72.14 \| \| --- \| \| 67.05 \| \| 64.33 \| \| 67.89 \| \| 71.44 \| \| 70.48 \| | \| 35.11 \| \| --- \| \| 30.47 \| \| 28.77 \| \| 26.45 \| \| 27.53 \| \| 23.47 \| |

Figure 1 E

| WT | PTS | DOX | DOX+PTS |
| --- | --- | --- | --- |
| \| 0.776 \| \| --- \| \| 0.745 \| \| 0.712 \| \| 0.697 \| \| 0.705 \| \| 0.798 \| | \| 0.803 \| \| --- \| \| 0.814 \| \| 0.8 \| \| 0.794 \| \| 0.745 \| \| 0.731 \| | \| 0.611 \| \| --- \| \| 0.596 \| \| 0.641 \| \| 0.612 \| \| 0.603 \| \| 0.579 \| | \| 0.672 \| \| --- \| \| 0.693 \| \| 0.684 \| \| 0.701 \| \| 0.689 \| \| 0.691 \| |

SOD

MDA

| WT | PTS | DOX | DOX+PTS |
| --- | --- | --- | --- |
| \| 0.596 \| \| --- \| \| 0.678 \| \| 0.711 \| \| 0.696 \| \| 0.811 \| \| 0.769 \| | \| 0.644 \| \| --- \| \| 0.513 \| \| 0.522 \| \| 0.603 \| \| 0.649 \| \| 0.715 \| | \| 1.413 \| \| --- \| \| 1.396 \| \| 1.445 \| \| 1.297 \| \| 1.375 \| \| 1.319 \| | \| 0.796 \| \| --- \| \| 0.745 \| \| 0.612 \| \| 0.547 \| \| 0.566 \| \| 0.613 \| |

Figure 1 F


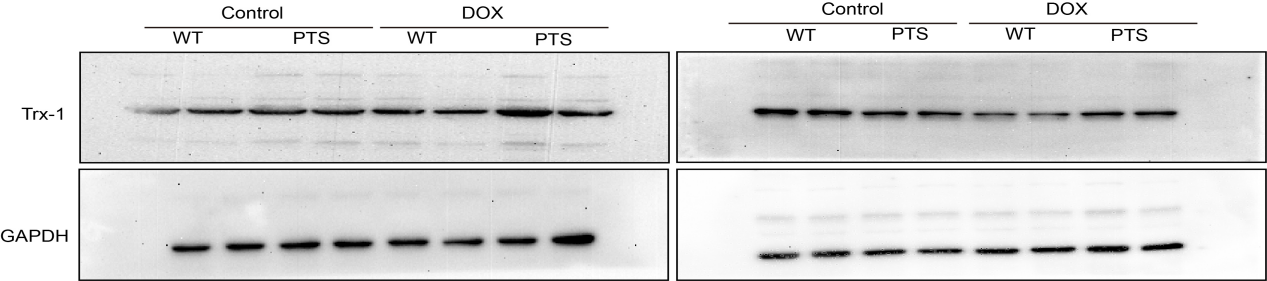


| WT | PTS | DOX | DOX+PTS |
| --- | --- | --- | --- |
| \| 0.996 \| \| --- \| \| 0.814 \| \| 0.877 \| \| 1.011 \| | \| 1.143 \| \| --- \| \| 1.222 \| \| 1.075 \| \| 1.167 \| | \| 0.783 \| \| --- \| \| 0.812 \| \| 1.098 \| \| 0.976 \| | \| 1.574 \| \| --- \| \| 1.612 \| \| 1.434 \| \| 1.476 \| |

Figure 2 A NLRP3 staining


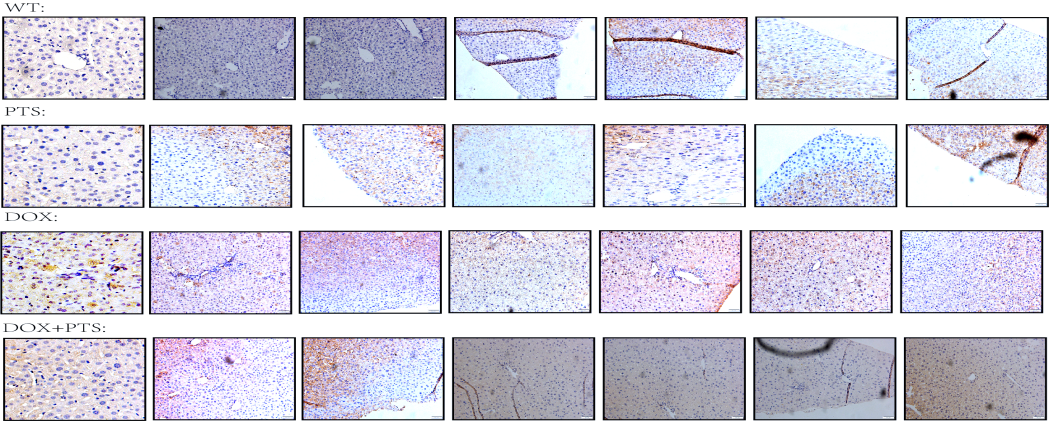


| WT | PTS | DOX | DOX+PTS |
| --- | --- | --- | --- |
| \| 1.033 \| \| --- \| \| 1.198 \| \| 0.976 \| \| 0.832 \| \| 0.911 \| \| 1.134 \| | \| 0.976 \| \| --- \| \| 0.894 \| \| 1.023 \| \| 1.011 \| \| 1.121 \| \| 0.945 \| | \| 1.877 \| \| --- \| \| 2.014 \| \| 1.763 \| \| 1.989 \| \| 2.134 \| \| 2.045 \| | \| 1.376 \| \| --- \| \| 1.245 \| \| 1.301 \| \| 1.211 \| \| 1.104 \| \| 1.044 \| |

Figure2 B

| WT | PTS | DOX | DOX+PTS |
| --- | --- | --- | --- |
| \| 1.144 \| \| --- \| \| 1.045 \| \| 0.977 \| \| 1.233 \| \| 1.055 \| \| 0.873 \| | \| 1.002 \| \| --- \| \| 0.976 \| \| 0.874 \| \| 1.123 \| \| 1.045 \| \| 0.932 \| | \| 5.445 \| \| --- \| \| 4.769 \| \| 5.132 \| \| 4.783 \| \| 5.078 \| \| 3.987 \| | \| 2.455 \| \| --- \| \| 3.126 \| \| 2.784 \| \| 3.102 \| \| 2.671 \| \| 2.545 \| |

NLRP3

| WT | PTS | DOX | DOX+PTS |
| --- | --- | --- | --- |
| \| 1.045 \| \| --- \| \| 1.011 \| \| 0.998 \| \| 0.976 \| \| 0.745 \| \| 0.873 \| | \| 1.132 \| \| --- \| \| 0.877 \| \| 0.912 \| \| 0.937 \| \| 1 \| \| 0.944 \| | \| 3.744 \| \| --- \| \| 3.545 \| \| 4.018 \| \| 3.277 \| \| 2.996 \| \| 3.155 \| | \| 1.576 \| \| --- \| \| 1.432 \| \| 1.457 \| \| 1.589 \| \| 1.712 \| \| 1.634 \| |

IL-1β

| WT | PTS | DOX | DOX+PTS |
| --- | --- | --- | --- |
| \| 0.969 \| \| --- \| \| 0.977 \| \| 0.834 \| \| 1.132 \| \| 1.002 \| \| 0.977 \| | \| 1.035 \| \| --- \| \| 0.749 \| \| 0.844 \| \| 0.941 \| \| 1.033 \| \| 1 \| | \| 3.245 \| \| --- \| \| 2.996 \| \| 3.011 \| \| 2.549 \| \| 2.083 \| \| 2.496 \| | \| 1.341 \| \| --- \| \| 1.469 \| \| 1.477 \| \| 1.518 \| \| 1.369 \| \| 1.601 \| |

IL-18

Figure2 C


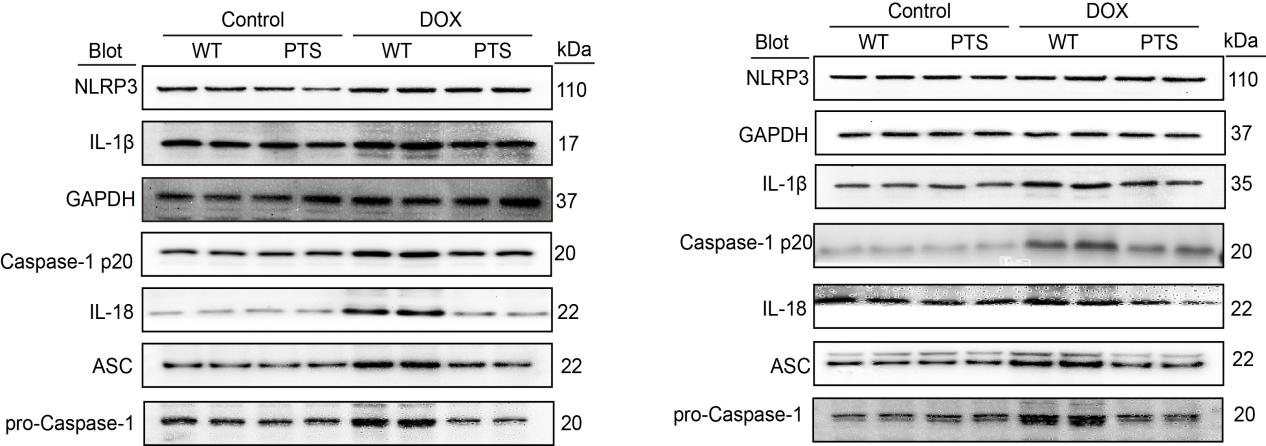


|  | WT | PTS | DOX | DOX+PTS |  |  | WT | PTS | DOX | DOX+PTS |
| --- | --- | --- | --- | --- | --- | --- | --- | --- | --- | --- |
| ASC | 0.998 | 1.023 | 2.567 | 1.344 |  | IL-18 | 1.003 | 0.932 | 3.445 | 1.677 |
|  | 0.975 | 1.045 | 2.988 | 1.569 |  |  | 0.989 | 1.005 | 3.756 | 1.543 |
|  | 1.013 | 0.978 | 3.045 | 1.432 |  |  | 1.013 | 0.944 | 4.012 | 1.436 |
|  | 1.044 | 0.945 | 2.798 | 1.233 |  |  | 0.956 | 1.002 | 3.798 | 1.512 |
| Cas-1 p20 | 0.996 | 0.945 | 3.044 | 1.034 |  | NLRP3 | 1.077 | 0.877 | 1.577 | 1.244 |
|  | 1 | 0.876 | 2.998 | 1.123 |  |  | 0.988 | 0.798 | 1.986 | 1.107 |
|  | 1.023 | 0.945 | 3.012 | 1.149 |  |  | 0.976 | 0.912 | 1.879 | 1.301 |
|  | 0.879 | 1.013 | 2.896 | 1.237 |  |  | 1.012 | 0.876 | 2.033 | 1.222 |
| IL-1β | 1.079 | 1.123 | 1.798 | 1.002 |  |  |  |  |  |  |
|  | 0.987 | 0.989 | 1.804 | 1.045 |  |  |  |  |  |  |
|  | 1.231 | 0.877 | 1.599 | 0.955 |  |  |  |  |  |  |
|  | 1.176 | 0.976 | 1.645 | 0.969 |  |  |  |  |  |  |

Figure 3A and B

DOX PTS+5 μM DOX

| 0 | 0.977 | 0.998 | 0.989 |
| --- | --- | --- | --- |
| 1 | 0.897 | 0.929 | 0.912 |
| 2 | 0.915 | 0.887 | 0.899 |
| 5 | 0.843 | 0.732 | 0.731 |
| 8 | 0.744 | 0.731 | 0.804 |
| 10 | 0.7077 | 0.752 | 0.817 |

| Control | 0.989 | 0.992 | 0.973 |
| --- | --- | --- | --- |
| 0 | 0.752 | 0.801 | 0.733 |
| 5 | 0.811 | 0.761 | 0.745 |
| 10 | 0.896 | 0.945 | 0.961 |
| 20 | 0.977 | 0.963 | 0.981 |

Figure4A and B

|  | Control | Trx-1 | DOX | DOX+Trx-1 |  |  | Control | Trx-1 | DOX | DOX+Trx-1 |
| --- | --- | --- | --- | --- | --- | --- | --- | --- | --- | --- |
| NOX-1 | 1.011 | 1.169 | 3.449 | 1.543 |  | NOX-4 | 0.733 | 0.899 | 3.447 | 1.545 |
|  | 0.945 | 0.977 | 3.667 | 1.801 |  |  | 1.045 | 1.034 | 4.012 | 1.333 |
|  | 0.879 | 1.132 | 3.976 | 1.371 |  |  | 0.998 | 0.845 | 3.796 | 1.432 |
|  | 1.113 | 0.833 | 2.798 | 1.451 |  |  | 1.132 | 0.877 | 3.554 | 1.256 |
|  | 0.956 | 0.879 | 3.496 | 1.512 |  |  | 0.979 | 0.993 | 4.121 | 1.369 |
|  | 1.178 | 1.077 | 3.577 | 1.203 |  |  | 0.981 | 0.927 | 4.132 | 1.132 |
|  |  |  |  |  |  |  |  |  |  |  |
|  | Control | Trx-1 | DOX | DOX+Trx-1 |  |  | Control | Trx-1 | DOX | DOX+Trx-1 |
| NLRP3 | 1.132 | 0.845 | 4.518 | 2.334 |  | IL-1β | 1.045 | 0.876 | 4.332 | 1.549 |
|  | 0.978 | 0.996 | 5.667 | 1.988 |  |  | 0.963 | 0.937 | 3.771 | 1.611 |
|  | 1.019 | 1.013 | 4.879 | 2.576 |  |  | 0.877 | 1.038 | 3.576 | 1.463 |
|  | 1.034 | 0.879 | 5.321 | 2.345 |  |  | 1.011 | 1.132 | 2.877 | 1.517 |
|  | 0.981 | 1.148 | 5.041 | 2.412 |  |  | 1.124 | 0.874 | 3.132 | 1.345 |
|  | 0.877 | 1.103 | 4.877 | 2.017 |  |  | 0.968 | 0.816 | 2.745 | 1.511 |

Figure4C


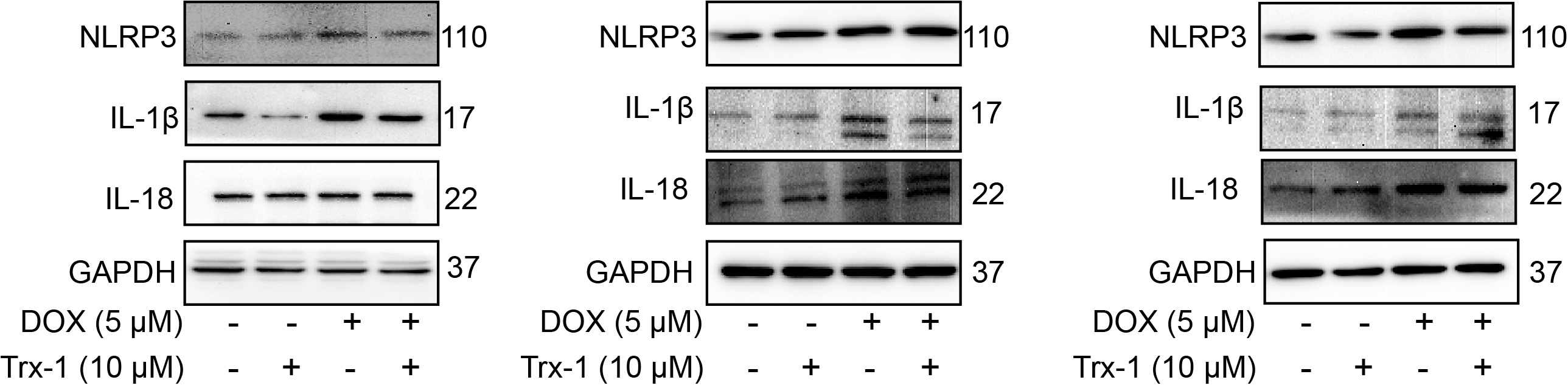


|  | Control | PTS | DOX | DOX+PTS |  |  | Control | PTS | DOX | DOX+PTS |
| --- | --- | --- | --- | --- | --- | --- | --- | --- | --- | --- |
| NLRP3 | 1.003 | 0.934 | 2.078 | 1.334 |  | IL-1β | 1.011 | 0.877 | 2.001 | 1.334 |
|  | 0.921 | 1.011 | 1.876 | 1.245 |  |  | 0.976 | 0.812 | 1.699 | 1.212 |
|  | 1.012 | 1.104 | 1.949 | 1.276 |  |  | 1.045 | 1.002 | 1.877 | 1.245 |
|  |  |  |  |  |  |  |  |  |  |  |
|  | Control | PTS | DOX | DOX+PTS |  |  |  |  |  |  |
| IL-18 | 1.01 | 0.944 | 1.544 | 1.012 |  |  |  |  |  |  |
|  | 0.998 | 1.005 | 1.579 | 0.994 |  |  |  |  |  |  |
|  | 0.976 | 0.933 | 1.496 | 1.103 |  |  |  |  |  |  |

Figure5A


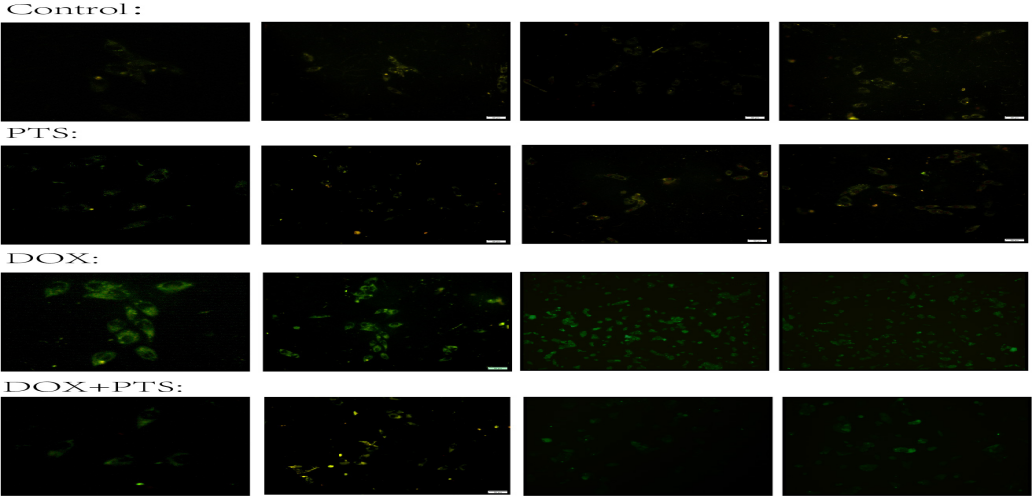


Figure5B

|  | Control | PTS | DOX | DOX+PTS |  |  | Control | PTS | DOX | DOX+PTS |
| --- | --- | --- | --- | --- | --- | --- | --- | --- | --- | --- |
| NOX1 | 1.078 | 0.869 | 3.877 | 1.543 |  | NOX-4 | 1.023 | 0.966 | 3.44 | 1.58 |
|  | 1.132 | 0.871 | 2.996 | 1.466 |  |  | 0.979 | 0.788 | 3.21 | 2.01 |
|  | 0.889 | 0.949 | 3.412 | 1.571 |  |  | 0.844 | 0.849 | 2.98 | 1.64 |
|  | 0.904 | 0.934 | 3.469 | 1.397 |  |  | 0.933 | 1.101 | 4.01 | 1.43 |
|  | 0.876 | 1.011 | 2.781 | 1.432 |  |  | 1.033 | 1.044 | 3.69 | 1.37 |
|  | 0.945 | 0.936 | 3.477 | 1.201 |  |  | 0.799 | 0.899 | 3.72 | 1.29 |

Figure5C

|  | Control | PTS | DOX | DOX+PTS |
| --- | --- | --- | --- | --- |
| Trx-1 | 1.007 | 0.979 | 0.312 | 0.877 |
|  | 0.998 | 1 | 0.335 | 0.912 |
|  | 0.896 | 0.944 | 0.249 | 0.845 |

Figure6A

|  | Control | PTS | DOX | DOX+PTS |  |  | Control | PTS | DOX | DOX+PTS |
| --- | --- | --- | --- | --- | --- | --- | --- | --- | --- | --- |
| NLRP3 | 1.044 | 0.877 | 4.431 | 1.541 |  | IL-1β | 1.132 | 0.867 | 3.142 | 2.013 |
|  | 0.998 | 0.912 | 3.976 | 1.372 |  |  | 1.044 | 0.912 | 3.111 | 1.477 |
|  | 0.877 | 0.849 | 4.102 | 1.421 |  |  | 0.998 | 0.894 | 4.002 | 1.571 |
|  | 0.932 | 1.066 | 3.541 | 1.507 |  |  | 0.877 | 1.105 | 2.978 | 1.496 |
|  | 1.145 | 1.004 | 3.704 | 1.345 |  |  | 0.846 | 1.004 | 3.169 | 1.431 |
|  | 1.021 | 0.935 | 3.198 | 1.277 |  |  | 0.945 | 1.012 | 3.547 | 1.541 |

Figure6B

|  | Control | PTS | DOX | DOX+PTS |  |  | Control | PTS | DOX | DOX+PTS |
| --- | --- | --- | --- | --- | --- | --- | --- | --- | --- | --- |
| NLRP3 | 1.01 | 0.96 | 2.78 | 1.23 |  | IL-1β | 1.11 | 0.877 | 2.044 | 1.345 |
|  | 0.97 | 0.84 | 3.1 | 1.16 |  |  | 0.94 | 0.932 | 2.512 | 1.421 |
|  | 0.83 | 0.88 | 2.88 | 1.3 |  |  | 0.921 | 1.011 | 2.344 | 1.307 |
|  |  |  |  |  |  |  |  |  |  |  |
|  | Control | PTS | DOX | DOX+PTS |  |  | Control | PTS | DOX | DOX+PTS |
| Cas-1 p20 | 1.01 | 0.84 | 2.84 | 1.21 |  | IL-18 | 0.978 | 0.82 | 2.031 | 1.044 |
|  | 0.96 | 0.92 | 2.55 | 1.04 |  |  | 1.033 | 0.781 | 2.115 | 1.132 |
|  | 0.87 | 0.95 | 2.61 | 0.99 |  |  | 0.845 | 0.766 | 2.132 | 1.201 |

Figure5C and Figure6B


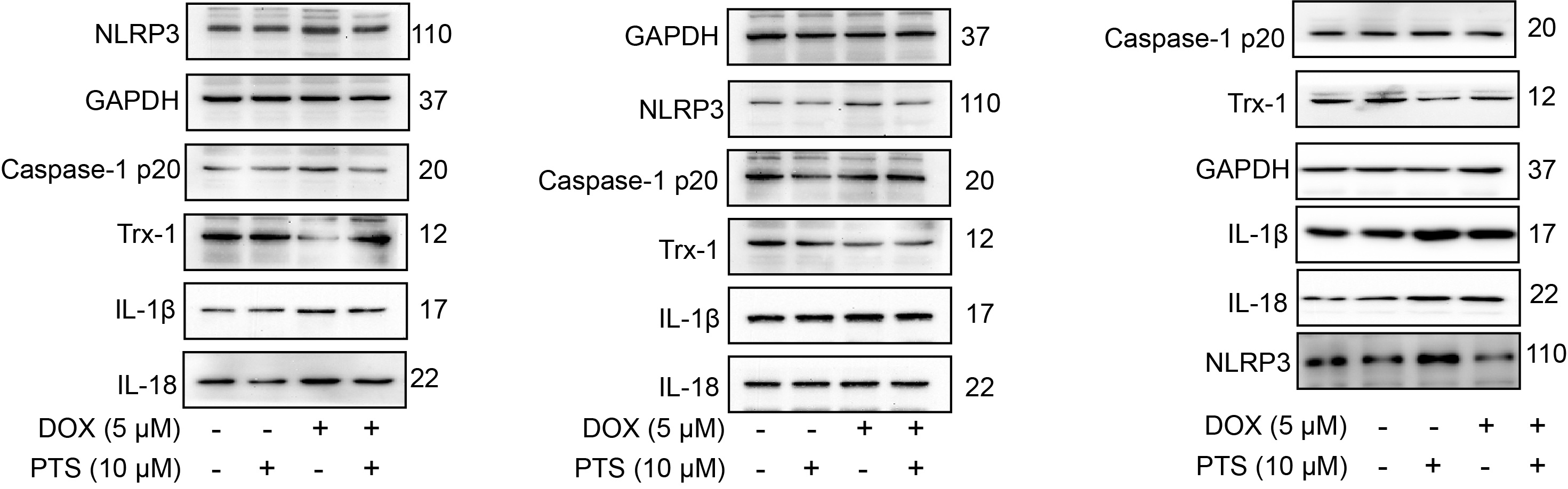

Supplement: Supplementary file 1 [file DataSheet1.DOCX]
